# Supplementary material for: Insights into cerebral haemodynamics and oxygenation utilising in vivo mural cell imaging and mathematical modelling
Source: Sci Rep. 2018 Jan 22;8:1373. doi: 10.1038/s41598-017-19086-z (PMC5778006; doi:10.1038/s41598-017-19086-z)
Supplement: Supplementary file 1 — Supplementary Material [file 41598_2017_19086_MOESM1_ESM.pdf]

Insights into cerebral haemodynamics and oxygenation utilising *in vivo* mural cell imaging and mathematical modelling - Supplementary Material

Paul W. Sweeney[1], Simon Walker-Samuel[2], and Rebecca J. Shipley[3]

[1,3]Mechanical Engineering, University College London

[2]Centre for Advanced Biomedical Engineering, University College London

[3]Correspondance: rebecca.shipley@ucl.ac.uk

Supplementary Tables

	Value	Units
No. of Segments	26662	-
Arterioles (SMA)	4.62	%
Capillaries (non-SMA)	91.90	%
Venules	3.46	%
No. of Nodes	26578	-
No. of Boundary Nodes	388	-
No. of Vessel O_2 Sources	26662	-
No. of Tissue O_2 Sinks	8000	-
Mean Diameter	5.26 ± 3.48	μm
Min. Diameter	3.00	μm
Max. Diameter	36.55	μm
Mean Length	1.75 ± 0.61	μm
Min. Length	0.13	μm
Max. Length	38.49	μm
Tissue Volume	$420 \times 420 \times 400$	μm^3
Vascular Density	2.63	%
Max. Extravascular Diffusion Distance	21.75	μm^{-1}

Table 1: Segmented network structural statistics.

Parameter	Value	Units	Reference
p_0	31	mmHg	[6,41]
Inlet H_D	0.45	-	[9]
$D\alpha$	6×10^{-10}	$cm^3 O_2$ cm^{-1} s^{-1} $mmHg^{-1}$	[31]
M_0	4.8	$cm^3 O_2$ $(100\ cm^3)^{-1}$ min^{-1}	Based on[32]
P_0	1	mmHg	[31,44]
C_0	0.5	$cm^3 O_2 cm^{-3}$	[19]
α_{eff}	3.1×10^{-5}	$cm^3 O_2 cm^{-3} mmHg^{-1}$	[19]
n	2.5	-	Based on[52]
P_{50}	40.4	mmHg	[52]
K	$9.3 \times 10^7 - 3 \times 10^8$	mmHg cm s/$cm^3 O_2$	[19,46,53]

Table 2: Assign simulation parameters and corresponding literature source.

Vessel Type	Pressure	Flow	Velocity	Wall Shear Stress	H_D
Arterioles	0.639	0.552	0.597	0.264	0.047
Capillaries	0.348	0.307	0.320	0.316	0.139
1	0.205	0.524	0.489	0.516	0.570
2	-0.065	0.550	0.171	0.331	0.490
3	-0.168	0.530	0.421	0.436	0.278
4	0.121	0.480	0.423	0.253	0.300
5	-0.262	0.350	0.328	0.290	0.261
6	-0.003	0.308	0.122	0.117	0.435
Venules	0.068	0.339	0.386	0.300	-0.171

Table 3: Pearson's correlation for each vessel type, including capillary orders 1 to 6, between PO_2 and fluid pressure, blood flow, blood velocity, vessel wall shear stress and vessel haematocrit. Data was compiled from the baseline simulation.

Scenario	Penetrating Arterioles	Precapillary Arterioles	Capillaries
1	53.0 ± 1.6	27.5 ± 2.8	1.3 ± 3.4
2	-	17.6 ± 6.6	-1.0 ± 8.3
3	28.3 ± 12.8	-5.2 ± 14.4	-9.6 ± 21.1
4	35.9 ± 12.4	-22.2 ± 16.9	-18.6 ± 25.3

Table 4: Percentage changes (mean ± SD %) from baseline velocity for each constriction scenario. Note, precapillary arteriolar data excludes the plasma skimming outlier as to not bias the data.

Scenario	Penetrating Arterioles	Precapillary Arterioles	Capillaries
1	-0.6 ± 1.0	-0.6 ± 2.2	1.1 ± 3.4
2	-	-8.5 ± 5.1	-2.9 ± 8.2
3	-16.7 ± 8.3	-26.2 ± 11.1	-11.4 ± 20.7
4	-11.7 ± 8.0	-39.5 ± 13.2	-20.2 ± 24.8

Table 5: Percentage changes (mean ± SD %) from baseline flow for each constriction scenario. Note, precapillary arteriolar data excludes the plasma skimming outlier as to not bias the data.

Scenario	Penetrating Arterioles	Precapillary Arterioles	Capillaries
1	-0.2 ± 0.01	-0.1 ± 0.3	-0.2 ± 0.3
2	-	-0.8 ± 0.9	-0.6 ± 0.9
3	-2.8 ± 2.9	-4.2 ± 2.4	-3.7 ± 2.1
4	-10.2 ± 5.1	-6.5 ± 2.5	-5.5 ± 2.3

Table 6: Percentage changes (mean ± SD %) from baseline pressure for each constriction scenario. Note, data for case 4 for penetrating arterioles is given by the unconstricted vessel segment situated next to the constricted vessel segment.

Scenario	Penetrating Arterioles	Precapillary Arterioles	Capillaries
1	-0.03 ± 0.2	-0.9 ± 1.4	0.2 ± 2.3
2	-	-2.4 ± 2.7	-1.6 ± 6.6
3	-4.9 ± 0.7	-4.7 ± 9.4	-6.0 ± 11.1
4	-2.7 ± 0.5	-9.0 ± 12.0	-11.6 ± 15.0

Table 7: Percentage changes (mean ± SD %) from baseline viscosity for each constriction scenario. Note, data for case 4 for penetrating arterioles is given by the unconstricted vessel segment situated next to the constricted vessel segment.

Supplementary Figures

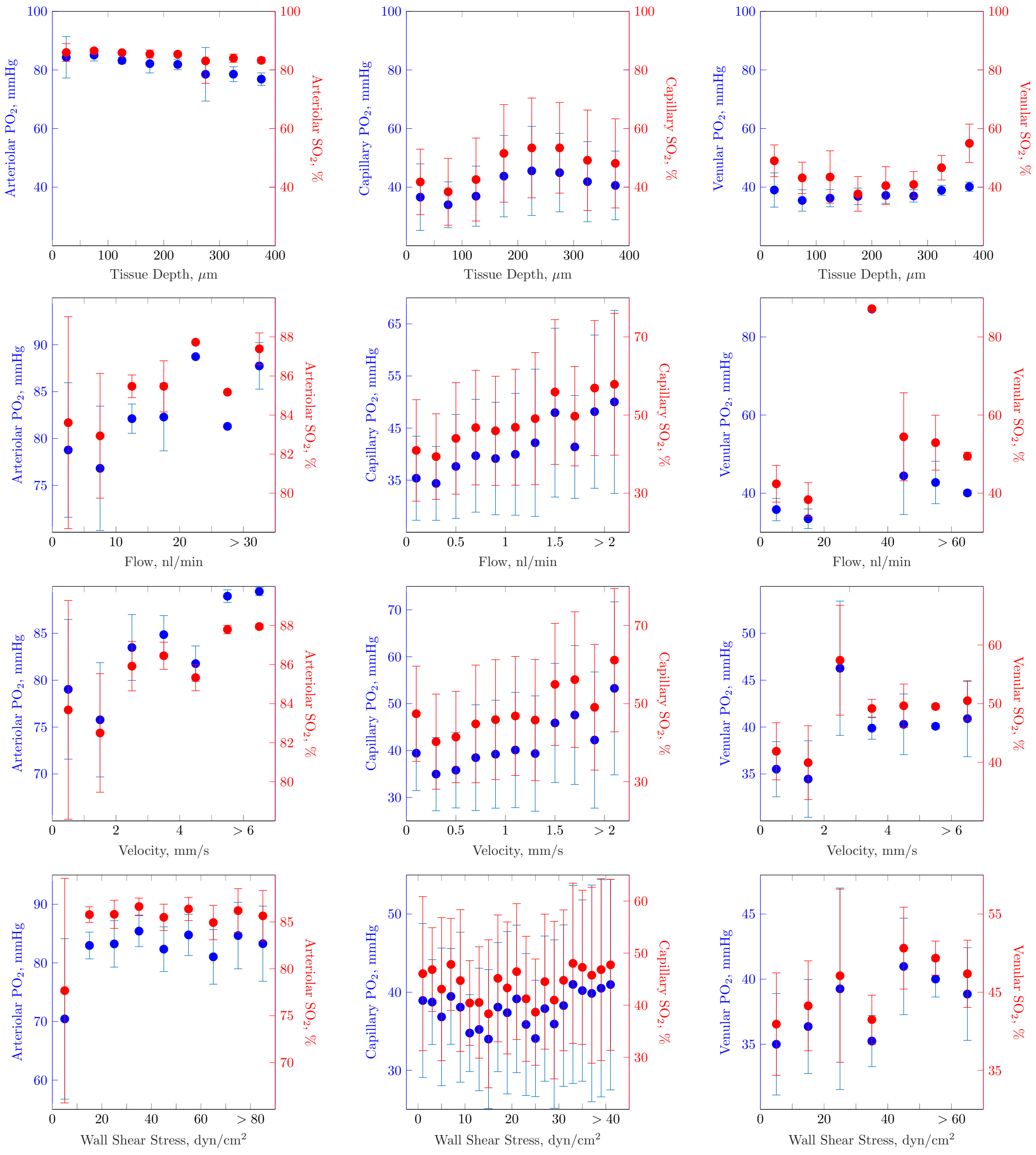

Figure 1: PO$_2$ and SO$_2$ versus tissue depth, flow, velocity and wall shear stress, for each vessel type. Data is expressed as mean ± standard deviation.

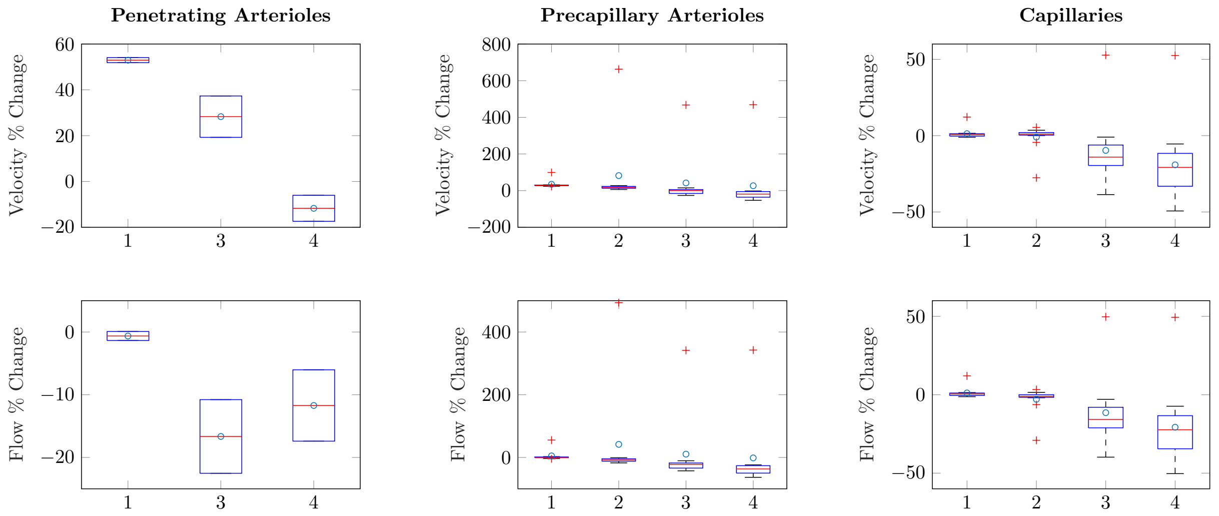

Figure 2: Box plots with outliers of blood velocity and flow changes in response to vasoconstriction. Precapillary arteriolar outlier exhibited a case of plasma skimming to containing RBCs post-constriction and so was excluded as to not bias results. The means for each case are indicated by circles and outliers (data larger than $q_3 + \frac{3}{2}(q_3 - q_1)$ or smaller than $q_1 - \frac{3}{2}(q_3 - q_1)$, where q_1 and q_3 are the 25th and 75th percentiles, respectively) by red crosses.

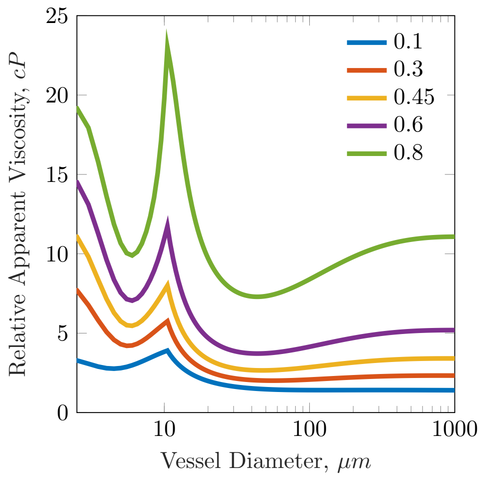

Figure 3: Apparent blood viscosity calculated using the empirical viscosity laws of Pries & Secomb[20].

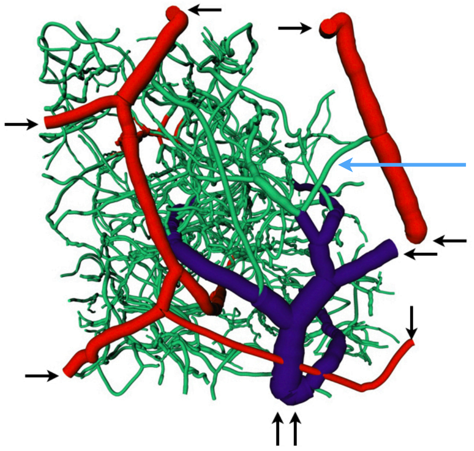

Figure 4: Birds-eye view of the cortical block. Arrows indicate the boundaries of the pial vessels in which pressure conditions were assigned. Vessel classifications are (red) arterioles, (green) capillaries and (blue) venules.The blue arrow indicates the first-order vascular shunt.

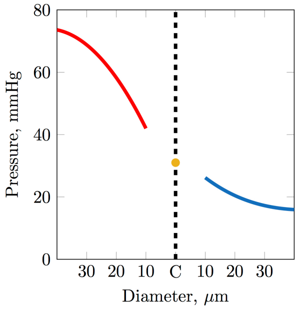

(a)

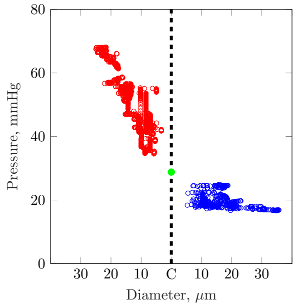

(b)

Figure 5: (Left) Arteriolar (red) and venula (blue) pressures as a function of diameter. C signifies the capillary cut-off, with the mean capillary pressure of 31 mmHg circled[6]. (Right) A scatter plot of baseline pressures for each vessel type calculated in this study.

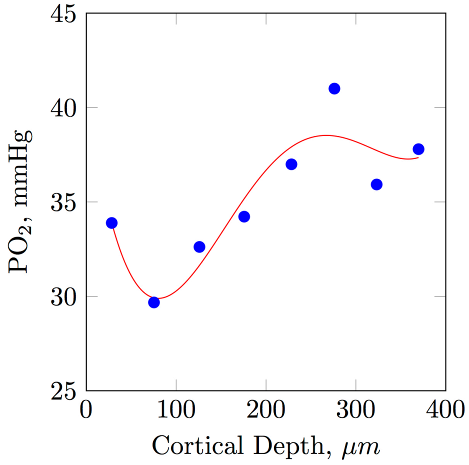

Figure 6: Data-fitted curve (red) of capillary PO_2 relative to cortical depth, along with the experimental data of Sakadžić *et al.*[22] (blue).

The Non-trivial Relationship Between Blood Velocity and Flow

Let V_i and Q_i represent the velocity and flow down a single vessel, where $i = 1, 2$ represents pre- and post-constriction, respectively. The relationship between V_i and Q_i is defined by Poiseuille's law such that

$$V_1 = \frac{Q_1}{\pi r_1^2} \quad \text{and} \quad V_2 = \frac{Q_2}{\pi r_2^2}, \tag{18}$$

where r_i is the radius of the vessel pre- and post-constriction.

If we assume the post-constriction velocity is greater that pre-constriction such that $V_1 < V_2$, then

$$\frac{Q_1}{\pi r_1^2} < \frac{Q_2}{\pi r_2^2} \Rightarrow Q_1 < \left(\frac{r_1}{r_2}\right)^2 Q_2. \tag{19}$$

Since we know $r_2 < r_1$ the term, $(r_1/r_2)^2$, is greater than one. This suggests we can obtain a post-constriction flow is smaller than the initial pre-constriction state, $Q_2 < Q_1$, thereby satisfying (19) where we have assumed blood velocity is greater post-constriction, $V_2 > V_1$. Fig. 7 shows all values for Q_2 in which a flow decrease and a velocity increase post-constriction can be found. Note, mean radii and blood flow values were used from the constriction of penetrating arterioles data.

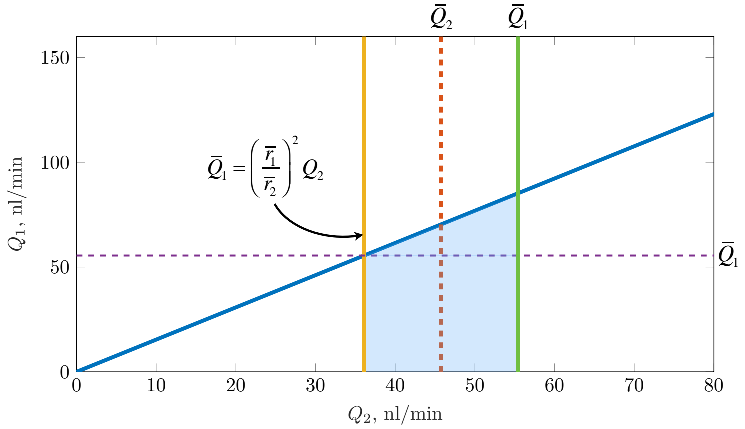

Figure 7: Example of flow decrease yet velocity increase in constricted vessel. Data on constriction of penetrating arterioles was used here, the hat notation indicates the mean value. All the points lying below the blue line satisfy the equation $\bar{r}_2^2 Q_1 < \bar{r}_1^2 Q_2$. The shaded blue area indicates all points satisfying the the conditions $V_1 < V_2$ and $Q_1 > Q_2$. That is to say, all flow values exhibiting a decrease post-constriction, yet an increase in blood velocity, relative to the pre-constriction state.
